# The principles of physical restraint use for hospitalized elderly people: an integrated literature review

**DOI:** 10.1186/s13643-021-01676-8

**Published:** 2021-05-01

**Authors:** Azam Sharifi, Narges Arsalani, Masoud Fallahi-Khoshknab, Farahnaz Mohammadi-Shahbolaghi

**Affiliations:** Nursing Department, Iranian Research Center on Aging, University of Social Welfare and Rehabilitation Sciences, Tehran, Iran

**Keywords:** Physical restraint, Integrative review, Principles, Elderly people

## Abstract

**Background:**

Physical restraint (PR) is a routine care measure in many hospital wards to ensure patient safety. However, it is associated with many different professional, legal, and ethical challenges. Some guidelines and principles have been developed in some countries for appropriate PR use. The present study aimed to explore the principles of PR use for hospitalized elderly people.

**Methods:**

This was an integrative review. For data collection, a literature search was conducted in Persian and English databases, namely Magiran, Scientific Information Database (SID), Scopus, Google Scholar, Web of Science, and PubMed as well as the websites of healthcare organizations and associations. Eligibility criteria were publication in English or Persian between January 1, 2010, and January 1, 2021, and description of the principles of PR use for hospitalized elderly people. The Preferred Reporting Items for Systematic Reviews and Meta-Analyses (PRISMA) statement was used for document screening and selection, while the critical appraisal tools of the Joanna Briggs Institute (JBI) and the Appraisal of Guidelines for Research and Evaluation II (AGREE II) instrument were used for quality appraisal. The data were analyzed through constant comparison.

**Results:**

Primarily, 772 records were retrieved, while only twenty were eligible for the study. The principles of PR use for hospitalized elderly people were categorized into six main categories, namely principles of education for PR use, principles of decision making for PR use, principles of implementing the PR procedure, principles of monitoring patients with PR, principles of PR use documentation, and principles of PR management.

**Conclusion:**

PR should be used only by trained healthcare providers, with the consent of patient or his/her family members, with standard devices and safe techniques, based on clear guidelines, and under close managerial supervision. Moreover, elderly people with PR should continuously be monitored for any PR-related complications. The findings of the present study can be used for developing clear PR-related guidelines.

**Supplementary Information:**

The online version contains supplementary material available at 10.1186/s13643-021-01676-8.

## Background

Medical and health-related advances in the second half of the twentieth century significantly improved life expectancy. Consequently, the global population is progressively aging so that estimates show the global aging population will reach two billions by 2050 [1–4]. Aging is associated with many different health-related problems. Most elderly people suffer from chronic illnesses such as stroke, cancer, diabetes mellitus, dementia, and cardiovascular disease. These problems require frequent hospitalizations [4–7].

Hospitalization is associated with many different adverse events and safety issues because most elderly people suffer from poor health status, cognitive impairments, and functional problems and, hence, are at risk for fall, removal of medical devices connected to the body, and injury to self and others [8–12]. In order to minimize these risks, healthcare providers often use physical restraint (PR) to limit patients’ body movements [13–15]. By definition, PR is “any action or procedure that prevents a person’s free body movement to a position of choice and/or normal access to his/her body by the use of any method that is attached or adjacent to a person’s body and that he/she cannot control or remove easily” [16]. These methods include belts (at the wrist, ankle, chest, waist), bedrails, and chairs [16, 17]. Evidence shows that PR is routinely used in hospitals [17–19] and elderly people receive PR during their hospital stay three times more than other hospitalized patients [9]. The prevalence of PR use for hospitalized elderly people is as high as 33–68% [7].

Although PR is used for safety purposes, studies show that its inappropriate use can endanger patient safety and cause serious physical and mental consequences. Its physical consequences include pressure ulcer, fracture, cardiac dysrhythmia, neuromuscular injuries, urinary and fecal incontinence, asphyxia, and strangulation-induced death [7, 8, 20–22]. The mental consequences of inappropriate PR use include anger, frustration, aggression, fear, humiliation, low self-confidence, delirium, depression, and anxiety [14, 17, 23]. Moreover, it is associated with ethical dilemmas and violates the autonomy and the respect for dignity principles of ethical practice [24, 25]. It also prolongs the length of hospital stay and increases the risk of fall and nosocomial infections [7, 22, 26]. Besides patients, healthcare providers are also at risk for the consequences of PR use. For instance, a study showed that while the prevalence of violence against healthcare providers was generally 4.5%, it significantly increased to 28% in case of PR use in pre-hospital settings [27]. PR use also causes negative feelings such as guilt and moral distress for healthcare providers [9, 13, 28].

Studies show that more than 80% of healthcare providers have limited knowledge and skills about appropriate PR use for hospitalized patients [20, 29–33]. Such lack of knowledge and skills not only causes serious problems for patients, but also causes professional, legal, and ethical challenges for healthcare providers. Therefore, clear guidelines are necessary for improving the quality of PR use and reducing its adverse consequences [23, 25, 34]. Several guidelines have so far been developed in this area. Most of these guidelines highlight the importance of maintaining patient’s autonomy, involving them in decision making about PR use, and minimizing PR use in healthcare settings [18, 35–38].

PR-related guidelines are context-bound, and hence, those which are appropriate for the needs, priorities, policies, and resources of certain contexts cannot be used in other sociocultural contexts [23, 39–41]. Review studies are needed to make appropriate conclusions and decisions about the principles of appropriate PR use and develop effective PR-related guidelines. Previous studies in this area explored the consequences of PR use [9, 42], its legal and ethical requirements [32, 42], the effectiveness of PR-related guidelines [12, 43], and the effectiveness of interventions for minimizing PR use [7, 22]. However, to the best of our knowledge, none of them comprehensively and systematically described the principles of appropriate PR use. Therefore, the present study was conducted to address this gap. The aim of the study was to explore the principles of PR use for hospitalized elderly people.

## Methods

This integrative review was conducted using the Whittemore and Knafl’s method [44, 45]. Initially, we performed a pilot review in order to determine the best literature review strategy for the study. The results of this pilot review revealed that the retrieved data were appropriate for integrative review. Integrative review is an approach which integrates different types of documents and a broad range of methodologies and summarizes available evidence in order to provide a deeper understanding about a given phenomenon. Moreover, it facilitates the integration of theoretical works on the subject of interest [44, 46]. This approach has five main stages, namely problem identification, literature search, data evaluation, data analysis, and presentation [45].

### Stage 1: Problem identification

The first stage of integrative review is the precise determination of the problem [45]. The present integrative review was conducted to answer the following research question, “What are the principles of PR use for hospitalized elderly people?”

### Stage 2: Literature search

The first and the second authors of the study independently searched the literature on the principles of PR use for hospitalized elderly people published from January 1, 2010, to January 1, 2021. The search was done in the following online databases, Magiran, Scientific Information Database (SID), Scopus, Google Scholar, Web of Science, and PubMed. Moreover, in order to review the grey literature, we searched library resources and the websites of healthcare organizations and associations (Additional file 1) and used the Google search engine to search the World Wide Web. To ensure the comprehensive assessment of the available literature, we also manually searched the reference lists of the retrieved documents. Literature search was performed based on the inclusion criteria shown in Table 1. Search keywords were extracted from the Medical Subject Heading (MeSH) and included “physical restraint,” “regulation,” “legislation,” “rule,” “principle,” “guideline,” “recommendation,” “standard,” “hospital,” “aged,” “elderly,” and “older adult”. Boolean operators “AND” and “OR” were also used. The search protocol was limited to the literature published in either Persian or English. A medical librarian validated the literature search strategy. An example of the literature search strategy is provided in the Additional file 2.

**Table 1:** Inclusion and non-inclusion criteria

**Inclusion criteria**	Open access empirical and theoretical studies; books; policies; statements; functional codes; standards; clinical guidelines Publication between January 1, 2010, and January 1, 2021 Description of the principles of PR use for hospitalized elderly people Publication in English or Persian language
**Non-inclusion criteria**	Specifically related to home care Specifically related to psychiatric care Specifically related to other age groups

### Stage 3: Data evaluation

Document screening and selection were performed using the Preferred Reporting Items for Systematic Reviews and Meta-Analyses (PRISMA) statement [47]. After excluding duplicate records, the first and the second authors independently assessed the titles and the abstracts of the retrieved documents for eligibility. In case of any uncertainty about the inclusion or the exclusion of any document, its full-text was assessed. Disagreements between the first and the second authors were resolved by the third author. Finally, the first and the second authors independently extracted data about the authors, countries of origin, publication dates, and aims of the studies as well as the principles of PR use for hospitalized elderly people from the included documents and documented in a data collection sheet. Disagreements about data extraction were resolved through discussion.

The first and the second authors independently performed quality appraisal using the critical appraisal tools of the Joanna Briggs Institute (JBI) [48] and the Appraisal of Guidelines for Research and Evaluation II (AGREE II) instrument [49]. Disagreements were resolved by the third author. According to the integrative review method [44, 46, 50], all included studies were included in the final analysis irrespective of their quality appraisal scores.

### Stage 4: Data analysis

In integrative review, collected data are ordered, categorized, and summarized [44]. Based on the study aim and the type of the included documents, the constant comparison method was used for data analysis. As a well-known method for data analysis, constant comparison helps systematically categorize the data. The method consists of data reduction, data display, data comparison, conclusion drawing, and verification. Constant comparison is an appropriate method for data analysis in integrative reviews due to the wide variety of the data included in the review from documents with diverse methodologies. In this method, extracted data are constantly compared and grouped according to their similarities and finally, findings are described and summarized in the form of categories or themes [44, 46, 51]. For data analysis, the first and the second authors reviewed the full text of each included document for several times, selected appropriate meaning units, and systematically categorized similar meaning units. Finally, their generated categories were assessed, revised, and approved by all members of the research team.

## Results

### Stage 5: Presentation

#### Characteristics of the documents

**Fig. 1:**
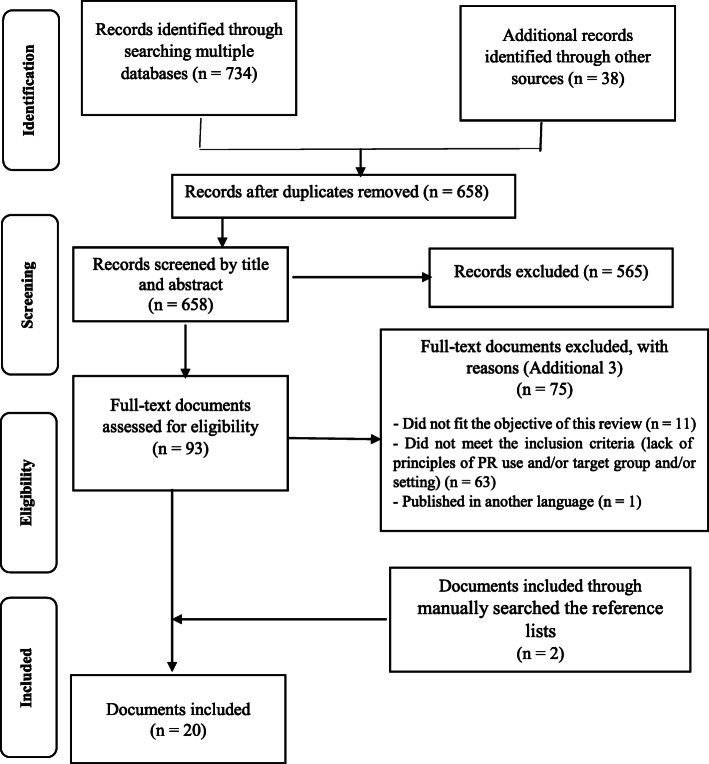
The PRISMA flow diagram of the study

Primarily, 772 documents were retrieved. After assessing the titles and the abstracts of these documents, 679 documents were excluded due to either duplication or irrelevance to the study aim. Finally, the full texts of the remaining 93 documents were assessed for eligibility; 75 documents were excluded due to ineligibility (Additional file 3). Finally, twenty eligible documents were included in the study. Figure 1 shows the PRISMA flowchart of the study.

All included documents were in English language. Additional file 4 presents the characteristics of the included documents, including data about the authors (individuals and/or organizations), countries of origin, publication dates, types of documents, aims of the studies, and quality scores. Included documents were four guidelines and sixteen journal articles, reports, expert consensus, books, statements, and policy papers which had been developed in the USA (*n* = 12), Canada (*n* = 3), Australia and New Zealand (*n* = 2), Ireland (*n* = 1), Turkey (*n* = 1), and Singapore (*n* = 1). The included documents were of moderate to high quality appraisal based on the critical appraisal tools (JBI and AGREE II) [48, 49].

#### Principles of PR use for hospitalized elderly people

**Fig. 2:**
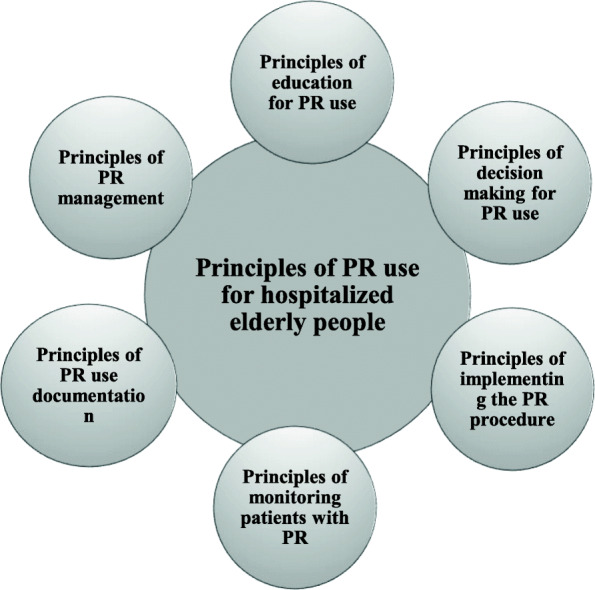
Visual presentation of findings

In integrative review, findings are described, summarized, and categorized [44, 51]. Accordingly, the principles of PR use for hospitalized elderly people were grouped into six main categories which are explained in the following. Figure 2 shows a visual presentation of the data.

#### Principles of education for PR use

One of the most important principles of PR use is to provide PR-related educations to all healthcare providers involved in PR use, including physicians, nurses, auxiliary nurses, and students [30, 35, 37, 43]. Healthcare providers should have adequate knowledge and skills for patient assessment, PR use, and PR outcome evaluation [30]. PR-related educational programs should focus on strategies for the effective management of high-risk situations in order to minimize the need for PR [13, 37]. These strategies include all PR alternatives which may vary according to the resources and equipment of the immediate environment. Moreover, educational programs should cover the principles of intelligent decision making about PR use, its legal and ethical requirements, the responsibilities of individuals involved in PR use, safe and proper PR-related techniques, appropriate physical and mental patient assessment, early diagnosis of PR-related consequences, and appropriate PR documentation [11, 32, 42]. PR-related educations should be provided during formal university education, at the time of recruitment to the profession, and during in-service continuous education programs through both face-to-face and online courses. In online courses, measures should be taken to clarify probable ambiguities and answer participants’ questions. PR-related educations can be provided periodically, for instance 1 h weekly or 6 h monthly [32]. The educational managers of hospitals should also keep a record of each healthcare provider’s participation in PR-related educational programs in his/her employment records [11, 32].

#### Principles of decision making for PR use

Before PR use, healthcare providers need to assess the intended patient, perform clinical examination, and take his/her complete medical history [9, 10]. In physical examination and history taking, patient’s physical and mental conditions and previous history of fall should carefully be assessed [32, 42]. Moreover, all factors which may cause confusion, wandering, agitation, and aggression should be determined and effectively managed [7, 8]. Unnecessary invasive procedures and connections should also be discontinued and removed as soon as possible because they can cause agitation [11, 36, 52].

There should be rational reasons for PR use [10, 12, 32]. The most important reasons for PR use for hospitalized elderly people are the risks of serious injury to self or others, fall, imbalance, and removal of life support connections (such as endotracheal tube, ventricular catheters, or arterial catheters) [37, 38, 42, 53]. PR should not be used for delirium management because immobility can increase the risk of agitation and injury [13, 54]. Moreover, PR should never be used in case of staff shortage and environmental unsuitability or for staff convenience and patient punishment [10, 37, 38, 52]. The risks associated with PR use should also be weighed against the risks of not using it [9, 13, 42]. In addition, PR should be used as the last resort for ensuring patient safety and when its advantages are greater than its disadvantages and associated risks [7, 35, 37, 43, 55]. Some studies recommended approaches and strategies for minimizing PR use which are shown in Table 2. Of course, some of these strategies may incur some costs. Healthcare providers should select and use the best strategies based on the immediate environment and sociocultural and economic conditions [7, 8, 18, 22].

**Table 2:** Approaches and strategies for minimizing PR use

Approaches	Strategies
Psychological support	Establishing relationship with patient and his/her family members to collect data about his/her daily habits and behaviors; increasing family members’ attendance at patient bedside; emotional support; stress management; coping enhancement
Physiological support	Removing unnecessary connections; fulfilling patient’s needs; pain management; medication management
Environmental modifications	Using motion sensors, alarms, low-low beds, and floor pads; reducing environmental stimuli; continuous patient monitoring
Managerial and organizational measures	Modifying organizational culture to reduce PR use; increasing nurse-patient ratio; promoting managerial supervision; providing healthcare providers with clinical guidelines; providing nurses and physicians with educations about PR use, its legal and ethical considerations, and its adverse consequences; and modifying PR-related attitudes

Another principle of decision making for PR use is to consider the opinions and preferences of patients, their surrogates, and their families. They have the right to know the reasons for PR use and its benefits and potential risks. Therefore, they should adequately be informed and their informed consent should be obtained [10–12, 32, 36, 37, 42]. Most studies highlighted that PR should be used only with medical order [10, 11, 36, 42]. In emergency situation which physicians may not be accessible, nurses can use PR without medical order and families’ consent but they should obtain medical order and inform family members about patients’ conditions as soon as possible [9, 11]. Some studies noted that besides physicians, registered nurses or medical assistants can also prescribe PR [32, 38]. Nonetheless, PR-related decisions should be made collaboratively and the opinions of all members of healthcare team should be considered [10, 11, 36, 42].

#### Principles of implementing the PR procedure

The devices and equipment for implementing the PR procedure should be standard, comfort, safe, and appropriate in size and should be made with soft and flexible materials, particularly foamed plastic or sheep skin. The PR procedure should never be implemented using clothes, bed sheets, or bandage. Moreover, PR-related devices and equipment should be equipped with locks which are easily opened or knots which are easily untied, should be made in various sizes, and should be used based on their user manuals [18, 36–38]. The PR procedure should also be implemented using safe and appropriate procedures [12, 18, 36–38, 56, 57]. The following points should be taken into account when implementing the PR procedure:

- Patient’s bed or chair should be locked and set at the lowest possible height.
- Patient should be positioned in proper body alignment in order to prevent neurovascular injuries.
- Primarily, devices should be used that apply the lowest level of immobility. Examples of these devices are gloves and elbow immobilizers.
- PR devices should be appropriate for patient size.
- The body surface on which PR is applied should be regularly assessed.
- PR should not be applied on catheters connected to the patient.
- At most two limbs should be restrained at any given time and the four limbs should never be restrained simultaneously.
- Bony prominences should be protected using pads in order to prevent tissue injury.
- PR devices should be connected to bed so that they freely move along with changes in the elevation of the head of bed.
- PR devices should never be fastened tightly because they may reduce blood flow to the limbs.
- PR devices should be fixed with knots which are easily untied so that they can easily be removed in emergency situations.

#### Principles of monitoring patients with PR

The monitoring of elderly people with PR should be performed using an individualized care plan and by registered nurses [10, 11, 35]. In this plan, patient’s physical and mental conditions should regularly be assessed every 15–30 min. In physical patient monitoring, the functions of the respiratory system (respiratory rate and rhythm), cardiovascular system (heart rate and rhythm, blood pressure, and capillary refilling), integumentary system (color, temperature, wound, edema), nervous system (sense and mobility of distal tissues and level of consciousness), and connected catheters (if any) should be carefully assessed. In mental patient monitoring, patient should be assessed for the symptoms of fear, humiliation, anger, and aggression [12, 32, 36–38]. Moreover, basic needs related to nutrition, hydration, hygiene, and elimination should be assessed and fulfilled [36, 37, 42, 52] based on the ethical principles of PR use including respect for self-esteem and privacy [7, 11, 32].

Some studies recommended the monitoring of elderly people under PR using closed-circuit television cameras or direct observation [35–37, 42]. Patient should be continuously monitored by registered nurses respecting any need for PR discontinuation [7, 11, 32]. PR should be used for a short period of time and discontinued as soon as patient’s need for PR is eliminated [7, 38, 42, 56]. Some studies reported that PR for elderly people should not be used for more than 4 h [37, 42]. During each 2-h course of PR use, PR should be removed for 10–15 min and patient’s response to it should be frequently assessed [7, 11, 32]. PR should immediately be removed in case of any PR-related complication [32, 37]. Healthcare providers should also monitor patients throughout the 24 h after PR removal respecting any PR-related complications or death [37].

#### Principles of PR use documentation

All PR-related care measures should clearly be documented in patient’s medical records [9–12, 35–37, 42]. PR-related documentation should include:

- Any unsuccessful measure for using PR alternatives to manage high-risk behaviors of the intended patient
- Reasons for PR use
- Medical order for PR with details about the time at which PR is applied and removed, the number of the restrained limbs, and the type of devices used for PR (Note: any PRN order for PR use should be avoided)
- The consent of patient, family members, or surrogate for PR use
- Patient’s physical and mental conditions before, during, and after PR use
- The process of physical and mental assessment before, during, and after PR use
- Patient’s response to PR
- Any injury or death from the beginning of PR use up to 24 hours after its removal

#### Principles of PR management

The management system of hospitals should develop plans and strategies for eliminating PR use. One of the principles of PR management is to support PR-free care using safety procedures [8, 11, 18, 30, 43]. In collaboration with healthcare providers, hospital managers should develop clear policies and guidelines for PR-related decision making and procedures. Such policies should be available in written format for all healthcare providers in healthcare settings [10, 37, 38]. Moreover, hospital managers should periodically assess all documents related to PR use for elderly people [37].

## Discussion

This study aimed to explore the principles of PR use for hospitalized elderly people. Twenty qualified documents were analyzed in the study. Findings revealed that these principles are related to education and decision making for PR use, implementing the PR procedure, monitoring patients with PR, documenting PR use, and managing PR use.

Adequate care-related knowledge is among the most basic requirements of any quality healthcare-related measure. Nonetheless, studies show that physicians and nurses have limited knowledge about PR use [18, 20, 31–33, 58]. A study showed that 85% of nurses and 90% of physicians had received no PR-related education [33]. Therefore, adequate quality education about the principles of appropriate PR use for elderly people should be provided to all healthcare providers. Such educations can reduce the use and the complications of PR [7, 58, 59]. Of course, pure education programs cannot guarantee safe PR-related practice [9, 23, 53] and adequate staffing, quality devices and equipment, and PR alternatives are needed to reduce the use and the complications of PR.

The second main category of the study was related to the principles of decision making for PR use. The most important point in making PR-related decisions is that PR should be considered as the last resort for ensuring patient safety. Nonetheless, evidence shows that PR is used as an accessible and routine option to reduce workload in case of staff shortage [11, 17, 20, 58–60], create a sense of security among healthcare providers, and even impose discipline or punishment on patients [7, 17, 60]. Such uses of PR contradict the principles of ethical practice. Studies show that PR-related decision making is affected by a wide range of factors such as care delivery environment, healthcare providers’ knowledge and attitude, managerial regulations, and cultural context [28, 53, 61–64]. All these factors should be taken into account while developing PR-related guidelines and protocols. Moreover, PR-related decisions should be made by a multidisciplinary team. In other words, the responsibility of PR use needs to be carried by all healthcare providers [10, 12]. However, some studies reported PR use as an independent nursing intervention, denoting that other healthcare providers do not participate in PR-related decision making [17, 32, 60, 61]. Therefore, PR-related complications can cause professional, legal, and ethical consequences for nurses. In order to prevent these consequences, all healthcare providers should be involved in decision making about PR use. We also found that PR should be used with the consent of the intended patient, his/her family members, or his/her surrogate. Contrary to this finding, some studies showed that PR is used without any consent [24, 31, 60, 65, 66]. Therefore, PR-related guidelines and protocols should include items on patient consent and hospital managers are recommended to exercise closer supervision in this area.

The third main category of the principles of PR use for elderly people was related to the principles of implementing the PR procedure. This procedure should be implemented using standard PR-specific devices. All healthcare providers should also receive adequate education about their appropriate use [30, 32, 35]. Nonetheless, a study reported the use of non-standard and inappropriate devices for PR and showed that PRs had been attached to side rails instead of bed frame in 91% of cases [60]. Such practice violates the principles of safe PR use because attaching PR devices to side rails may cause serious injuries to the restrained limb in case of the sudden fall of side rails. Another study also showed that 86.7% of nurses had poor PR-related practice [58]. Evidence showed that using non-standard devices and unsafe techniques for PR can cause patients injuries and even death [21, 32, 60]. Therefore, hospital managers and authorities are recommended to provide healthcare providers with standard devices and clear guidelines for PR use.

Principles of monitoring patients with PR were the fourth main category of the study. Physical and mental monitoring of elderly people with PR should be performed using a regular, comprehensive, and individualized care plan. A study found that for most patients, PR is not immediately removed after agitation is managed and is continued without any rational reason [67]. Another study reported that in 46.9% of cases, nurses did not regularly perform skin assessment [68] and the most common complications related to inappropriate patient monitoring were agitation (72%) and impaired skin integrity (55.9%) [20]. Evidence shows that poor monitoring of patients with PR can negatively affect patient safety, increase the likelihood of medical errors, and cause different complications [7, 10, 13, 58, 60]. Therefore, all healthcare providers, particularly nurses, are recommended to pay more careful attention to patient monitoring during PR use.

The fifth main category of the study was related to the principles of PR documentation. Documentation of therapeutic and caring measures in patients’ medical records is a professional and legal responsibility of all healthcare providers, and patients’ medical records are a valuable source for safe and quality care delivery [69]. However, evidence shows that more than 70% of healthcare providers do not document PR use [58, 60, 66, 70]. A study showed that most physicians did not document PR-related orders in patients’ medical records. Consequently, nurses also avoid PR documentation due to their fear over the legal consequences of PR use [60]. Lack of PR documentation in patients’ medical records results in the lack of any evidence for assessing the quality and the complications of PR use. Therefore, quality education and managerial supervision are needed to promote PR documentation.

The six main category of the study was the principles of PR management. Hospital management system should develop plans to reduce PR use and support PR-free care. Evidence shows that clear PR-related guidelines and protocols can reduce healthcare providers’ uncertainties over PR use and improve care quality [18, 58, 66]. Moreover, regular managerial supervision of healthcare providers’ PR-related practice is among the major factors contributing to quality care. Lack of such supervision can result in increased use of PR [23, 60, 63]. Establishment of committees on PR use in hospitals is recommended in order to promote PR-free care and supervise healthcare providers’ PR-related practice.

## Limitations

One of the limitations of this study was our limited access to some databases. Moreover, this study only reviewed studies published in English and Persian. Although all documents included in the study had acceptable quality, the results of the present study should be used and generalized to other settings cautiously.

## Conclusion

PR is a high-risk and complex care measure and, hence, should be used based on clear principles and guidelines. This study suggests that all healthcare providers should receive quality PR-related education, make PR-related decisions in collaboration with patients and their family members, use standard and safe devices and techniques for PR, continuously monitor patients with PR, and carefully document all PR-related care measures in patients’ medical records. Moreover, managers and authorities in all hospitals should provide healthcare providers with clear guidelines for using PR. The findings of the present study can be used for developing culturally-appropriate PR-related guidelines and can be used in nursing research, practice, education, and management.

## Supplementary Information

**Additional file 1.** Websites of health care organizations and associations. Websites of health care organizations and associations included in the search.

**Additional file 2.** An example of the literature search strategy. An Example of search strategy used in the literature database of PubMed.

**Additional file 3.** List of excluded documents. List of excluded documents after full-text eligibility assessment.

**Additional file 4.** Characteristics of the included documents. The characteristics of documents included in the present study.

## Data Availability

The datasets used and/or analyzed during the current study are available from the corresponding author on reasonable request.

## Abbreviations

PR: Physical restraint
SID: Scientific Information Database
MeSH: Medical Subject Heading
PRISMA: Preferred Reporting Items for Systematic Reviews and Meta-Analyses
JBI: Joanna Briggs Institute
AGREE: Appraisal of Guidelines for Research and Evaluation
