# Supplementary material for: The principles of physical restraint use for hospitalized elderly people: an integrated literature review
Source: Syst Rev. 2021 May 1;10:129. doi: 10.1186/s13643-021-01676-8 (PMC8088072; doi:10.1186/s13643-021-01676-8)
Supplement: Supplementary file 1 — Additional file 1. Websites of health care organizations and associations. Websites of health care organizations and associations included in the search. [file 13643_2021_1676_MOESM1_ESM.docx]

**Additional file 1.** Websites of health care organizations and associations. Websites of health care organizations and associations included in the search.

| **Health care organizations and associations** | **Links** |
| --- | --- |
| ANA - American Nurses Association | http://www.nursingworld.org |
| AGS - The American Geriatrics Society | http://www.americangeriatrics.org |
| G-I-N - Guidelines International Network | http://www.g-i-n.net |
| SIGN - Scottish Intercollegiate Guidelines Network | http://www.sign.ac.uk |
| JBI - The Joanna Briggs Institute | http://www.joannabriggs.edu.au |
| AGREE - Appraisal of Guidelines for Research & Evaluation | http://www.agreetrust.org |
| NICE - National Institute for Health and Clinical Excellence | http://www.nice.org.uk |
| RCN - Royal College of Nursing | http://www.rcn.org.uk |
| NIH - National Institutes of Health | http://www.nih.gov |
| JCAHCO - Joint Commission on Accreditation of Healthcare Organizations | http://www.jointcommission.org |
| WHO - World Health Organisation | http://www.who.int |
| CDC - Centers for Disease Control and Prevention | http://www.cdc.gov |
| FDA - U.S. Food & Drug Administration | http://www.fda.gov |
| ICN - International Council of Nurses | http://www.icn.ch |
| NZGG- New Zealand Guidelines Group | [http://www.nzgg.org.nz](http://www.nzgg.org.nz/) |
| CADTH- Canadian Agency for Drugs and Technologies in Health | [http://cadth.ca](http://cadth.ca/) |
| CMA- Canadian Medical Association Infobase: Clinical & Practice Guidelines | http://www.cma.ca |
| Clinical Evidence | [http://clinicalevidence.bmj.com](http://clinicalevidence.bmj.com/) |
| College of Nurses of Ontario: Standards: | [http://www.cno.org](http://www.cno.org/) |
| RNAO- Registered Nurses Association of Ontario | <http://www.rnao.org> |
| British Columbia Council on Clinical Practice Guidelines | http://www.bcguidelines.ca |
| Hartford Institute for Geriatric Nursing | https://hign.org |
| Irish Nurses Association | http://www.inmo.ie |
| American Family Physician | https://www.aafp.org |
| Iran Ministry of Health and Mdical Education | <https://medcare.behdasht.gov.ir> |
| INO- Iranian Nursing Organization | http://ino.ir |
