# Supplementary material for: The principles of physical restraint use for hospitalized elderly people: an integrated literature review
Source: Syst Rev. 2021 May 1;10:129. doi: 10.1186/s13643-021-01676-8 (PMC8088072; doi:10.1186/s13643-021-01676-8)
Supplement: Supplementary file 2 — Additional file 2. An example of the literature search strategy. An Example of search strategy used in the literature database of PubMed. [file 13643_2021_1676_MOESM2_ESM.docx]

**Additional file 2.** An example of the literature search strategy. An Example of search strategy used in the literature database of PubMed.

| Search | Query | Results |
| --- | --- | --- |
| #15 | Search: **(((("physical restraint*"[Title/Abstract]) OR (Restraint, Physical[MeSH Terms])) OR ("mechanical restraint*"[Title/Abstract])) OR (‘‘physical immobilization’’[Title/Abstract])) AND (((((((("regulation*"[Title/Abstract]) OR ("legislation*"[Title/Abstract])) OR ("rule*"[Title/Abstract])) OR ("principle*"[MeSH Terms])) OR ("instruction*"[Title/Abstract])) OR ("guideline*"[MeSH Terms])) OR ("recommendation*"[Title/Abstract])) OR ("protocol*"[Title/Abstract]))** Filters: **Humans, English, Persian, Aged: 65+ years, from 2010/1/1 - 2021/1/1** | 57 |
| #14 | Search: **((((((("regulation*"[Title/Abstract]) OR ("legislation*"[Title/Abstract])) OR ("rule*"[Title/Abstract])) OR ("principle*"[MeSH Terms])) OR ("instruction*"[Title/Abstract])) OR ("guideline*"[MeSH Terms])) OR ("recommendation*"[Title/Abstract])) OR ("protocol*"[Title/Abstract])** Filters: **Humans, English, Persian, Aged: 65+ years, from 2010/1/1 - 2021/1/1** | 90,268 |
| #13 | Search: **((("physical restraint*"[Title/Abstract]) OR (Restraint, Physical[MeSH Terms])) OR ("mechanical restraint*"[Title/Abstract])) OR (‘‘physical immobilization’’[Title/Abstract])** Filters: **Humans, English, Persian, Aged: 65+ years, from 2010/1/1 - 2021/1/1** | 400 |
| #12 | Search: **"recommendation*"[Title/Abstract]** Filters: **Humans, English, Persian, Aged: 65+ years, from 2010/1/1 - 2021/1/1** | 19,197 |
| #11 | Search: **"instruction*"[Title/Abstract]** Filters: **Humans, English, Persian, Aged: 65+ years, from 2010/1/1 - 2021/1/1** | 4,413 |
| #10 | Search: **"protocol*"[Title/Abstract]** Filters: **Humans, English, Persian, Aged: 65+ years, from 2010/1/1 - 2021/1/1** | 35,845 |
| #9 | Search: **"guideline*"[MeSH Terms]** Filters: **Humans, English, Persian, Aged: 65+ years, from 2010/1/1 - 2021/1/1** | 12,753 |
| #8 | Search: **"principle*"[MeSH Terms]** Filters: **Humans, English, Persian, Aged: 65+ years, from 2010/1/1 - 2021/1/1** | 1,077 |
| #7 | Search: **"rule*"[Title/Abstract]** Filters: **Humans, English, Persian, Aged: 65+ years, from 2010/1/1 - 2021/1/1** | 7,149 |
| #6 | Search: **"legislation*"[Title/Abstract]** Filters: **Humans, English, Persian, Aged: 65+ years, from 2010/1/1 - 2021/1/1** | 796 |
| #5 | Search: **"regulation*"[Title/Abstract]** Filters: **Humans, English, Persian, Aged: 65+ years, from 2010/1/1 - 2021/1/1** | 15,376 |
| #4 | Search: **"mechanical restraint*"[Title/Abstract]** Filters: **Humans, English, Persian, Aged: 65+ years, from 2010/1/1 - 2021/1/1** | 19 |
| #3 | Search: **‘‘physical immobilization’’[Title/Abstract]** Filters: **Humans, English, Persian, Aged: 65+ years, from 2010/1/1 - 2021/1/1** | [1](https://pubmed.ncbi.nlm.nih.gov/?term=%22physical+restraint%2A%22%5BTitle%2FAbstract%5D+OR+%22mechanical+restraint%2A%22%5BTitle%2FAbstract%5D&filter=dates.2010%2F1%2F1-2021%2F1%2F1&filter=lang.english&filter=lang.persian&filter=age.aged&filter=hum_ani.humans&sort=relevance) |
| #2 | Search: **Restraint, Physical[MeSH Terms]** Filters: **Humans, English, Persian, Aged: 65+ years, from 2010/1/1 - 2021/1/1** | 325 |
| #1 | Search: **"physical restraint*"[Title/Abstract]** Filters: **Humans, English, Persian, Aged: 65+ years, from 2010/1/1 - 2021/1/1** | 202 |
