# Supplementary material for: The principles of physical restraint use for hospitalized elderly people: an integrated literature review
Source: Syst Rev. 2021 May 1;10:129. doi: 10.1186/s13643-021-01676-8 (PMC8088072; doi:10.1186/s13643-021-01676-8)
Supplement: Supplementary file 3 — Additional file 3. List of excluded documents. List of excluded documents after full-text eligibility assessment. [file 13643_2021_1676_MOESM3_ESM.docx]

**Additional file 3.** List of excluded documents. List of excluded documents after full-text eligibility assessment.

Ang SY, Bakar Aloweni FA, Perera K, et al. Physical restraints among the elderly in the acute care setting: Prevalence, complications and its association with patients’ characteristics. Proceedings of Singapore Healthcare. 2015;24(3):137-143. Reason for exclusion: Lack of principles of PR use for hospitalized elderly people.

Aydin ozkan S, Karaka T, Ister E. Validity and reliability of the perceptions of
restraint use questionnaire for use in Turkey. Turk Geriatr Derg. 2017;20(1):
30–7. Reason for exclusion: The objective of this primary study did not match review objective.

Bai X, Kwok TCY, Ip IN, Woo J, Chui MYP, Ho FKY. Physical restraint use and older patients’ length of hospital stay. Health Psychol Behav Med. 2014;2(1):160–70. Reason for exclusion: Lack of principles of PR use for hospitalized elderly people.

Azizpour M, Moosazade M, Esmaeili R. Use of physical restraints in Intensive Care Unit: A systematic review study, Acta Medica Mediterranea, 2017; 33: 129. Reason for exclusion: The objective of this study did not match review objective.

Bleijlevens MH, Wagner LM, Capezuti E, Hamers JP; International Physical Restraint Workgroup. Physical Restraints: Consensus of a Research Definition Using a Modified Delphi Technique. J Am Geriatr Soc. 2016;64(11):2307-2310. Reason for exclusion: The objective of this study did not match review objective.

Calvo-Francés F, Capezuti EA. The use of physical restraints in long-term care in Spain: A multi-center cross-sectional study. [BMC Geriatr](https://www.ncbi.nlm.nih.gov/pmc/articles/PMC5251344/). 2017; 17: 29. Reason for exclusion: Lack of principles of PR use for hospitalized elderly people.

[Chiba](https://www.researchgate.net/profile/Yumi_Chiba) Y, [Yamamoto-Mitani](https://www.researchgate.net/profile/Noriko_Yamamoto-Mitani) N, [Kawasaki](https://www.researchgate.net/scientific-contributions/Maki-Kawasaki-71235210) M. A national survey of the use of physical restraint in long-term care hospitals in Japan. Journal of Clinical Nursing 2012, 21, 1314–1326. Reason for exclusion: Lack of principles of PR use for hospitalized elderly people.

Fariña-López E, Estévez-Guerra GJ, Polo-Luque ML, Pogrányivá AH, Penelo E. Physical restraint use with elderly patients: perceptions of nurses and nursing assistants in Spanish acute care hospitals. Nurs Res. 2018;67(1):55–9. Reason for exclusion: Lack of principles of PR use for hospitalized elderly people in the context of this study.

Goethals S, De Casterlé BD, Gastmans C. Nurses’ ethical reasoning in cases
of physical restraint in acute elderly care: a qualitative study. Med Health Care Philos. 2013;16(4):983–91. Reason for exclusion: Lack of principles of PR use for hospitalized elderly people in the context of this study.

Barr J, Fraser GL, Puntillo K, Ely EW, Gélinas C, Dasta JF, et al. Clinical practice guidelines for the management of pain, agitation, and delirium in adult patient’s in the intensive care unit. Crit Care Med 2013;41(1):263—306. Reason for exclusion: The context of this study did not match the inclusion criteria.

Hou IC, Chen J, Lin FL, Yu HZ, Huang SF, Huang YJ. The evaluation of integrating physical restraint report and care record system. Studies in Health Technology and Informatics 2013, 192, 934*.* Reason for exclusion: The context of the document did not match the inclusion criteria.

Alkilany M. [Physical Restraint Among Older Adults an Argumentive Essay](https://papers.ssrn.com/sol3/papers.cfm?abstract_id=2782882). SSRN Electronic Journal. 2016. Reason for exclusion: Lack of principles of PR use for hospitalized elderly people.

Ben Natan M, Akrish O, Zaltkina B, Noy RH. Physically restraining elder residents of long-term care facilities from a nurses’ perspective**.** International Journal of Nursing Practice 2010; 16: 499–507. Reason for exclusion: Lack of principles of PR use for hospitalized elderly people.

Boustani MA, Campbell NL, Khan BA, Abernathy G, Zawahiri M, Campbell T, Tricker J, Hui SL, Buckley JD, Perkins AJ, Farber MO, Callahan CM. Enhancing care for hospitalized older adults with cognitive impairment: a randomized controlled trial. J Gen Intern Med. 2012 May;27(5):561-7. Reason for exclusion: Lack of principles of PR use for hospitalized elderly people in the context of this study.

American Geriatrics Society 2015 Beers Criteria Update Expert Panel. American Geriatrics Society 2015 Updated Beers Criteria for Potentially Inappropriate Medication Use in Older Adults. J Am Geriatr Soc. 2015;63(11):2227-46. Reason for exclusion: Lack of principles of PR use for hospitalized elderly people.

Chang, YY, Yu HH., Loh EW, Chang LY.The efficacy of an in-service education program designed to enhance the effectiveness of physical restraints. The Journal of Nursing Research, 2016, 24(1), 79-86. Reason for exclusion: The objective of this study did not match review objective.

Chou Y, Hsu YH; Wang YC; Chu CS; Liao MC. Chen LK. The adverse effects of physical restraint use among older adult patients admitted to the internal medicine wards: A hospital-based retrospective cohort study. The Journal of Nutrition, Health & Aging,  2020, 24(2):160-165. Reason for exclusion: Lack of principles of PR use for hospitalized elderly people in the context of this study.

Barton-Gooden A, Dawkins P and Bennett J. Physical restraint usage at a teaching hospital: A pilot study. Clin Nurs Res 2013; 1–18. Reason for exclusion: The context of this study did not match the inclusion criteria.

Berzlanovich AM, Schopfer J, Keil W. Deaths due to physical restraint. Dtsch Arztebl Int. 2012;109(3):27–32. Reason for exclusion: The objective of this study did not match review objective.

Crutchfield P, Gibb TS, Redinger MJ, Ferman D, Livingstone J. The conditions for ethical application of restraints. Chest. 2019; 155 (3): 617–25. Reason for exclusion: Target group did not match inclusion criteria.

Eltaliawi AG, El-Shinawi M, Comer A, Hamazah S, Hirshon JM. Restraint use among selected hospitalized elderly patients in Cairo, Egypt. BMC Res Notes. 2017;10(1):633. Reason for exclusion: Lack of principles of PR use for hospitalized elderly people in the context of this study.

Enns E, Rhemtulla R., Ewa V, Fruetel K, Holroyd-Leduc JM. A controlled quality improvement trail to reduce the use of physical restraints in older hospitalized adults. Journal of the American Geriatrics Society 2014, 62, 541-545. Reason for exclusion: Lack of principles of PR use for hospitalized elderly people in the context of this study.

Fariña-López E, Estévez-Guerra GJ, Gandoy-Crego M, Polo-Luque LM,
Gómez-Cantorna C, Capezuti EA. Perception of spanish nursing staff on the
use of physical restraints. J Nurs Scholarsh. 2014;46(5):322–30. Reason for exclusion: Lack of principles of PR use for hospitalized elderly people in the context of this study.

Goethals S, Casterlé BD, Gastmans C. Nurses’ decision-making process in
cases of physical restraint in acute elderly care: a qualitative study. Int J Nurs Stud. 2013;50(5):603–12. Reason for exclusion: Lack of principles of PR use for hospitalized elderly people in the context of this study.

Hevener S, Rickabaugh B, Marsh T. Using a decision wheel to reduce use
of restraints in a medical-surgical intensive care unit. Am J Crit Care. 2016;
25(6):479–86. Reason for exclusion: The objective of this study did not match review objective.

Huang HC, Huang YT, Lin KC, Kuo YF. Risk factors associated with physical restraints in residential aged care facilities: a community-based epidemiological survey in Taiwan. J Adv Nurs. 2014;70(1):130-43. Reason for exclusion: Lack of principles of PR use for hospitalized elderly people.

Gunawardena R, Smithard DG. The attitudes towards the use of restraint and restrictive intervention amongst healthcare staff on acute medical and frailty wards—a brief literature Review. Geriatrics 2019, 4, 50. Reason for exclusion: Lack of principles of PR use for hospitalized elderly people.

Guvercin CH, Samur M, Gurkan K. The other side of the coin: nurses’ views and behavior on physical restraint. Acta Bioethica. 2018;24(2):253–64. Reason for exclusion: The objective of this study did not match review objective.

Kandeel NA, Attia AK. Physical restraints practice in adult intensive care units in Egypt. Nursing & health sciences. 2013;15(1):79-85. Reason for exclusion: The context of the document did not match the inclusion criteria.

Hooseinrezaee H, Nouhi E, Taher harikandee S. The effect of education on trauma critical care nurses attitudes towards and knowledge and practices from the viewpoint of their about application of physical restraint. Journal of Nursing Education. 2015;4(1):31-8. (Persian) Reason for exclusion: Lack of principles of PR use for hospitalized elderly people.

Jafari KS, Danaii Kh, Dolatshahi P, Ramezani M., Roohparvar R., Sabaghiyan Peirov A. Hospital Accreditation Standards in Iran. Text in persian. 2010. (Persian) Reason for exclusion: Lack of principles of PR use for hospitalized elderly people.

Hughes L, Lane P. Use of physical restraint: ethical, legal and political issues. [Learning Disability Practice](https://www.researchgate.net/journal/Learning-Disability-Practice-1465-8712) , 2016: 19(4):23-27. Reason for exclusion: Target group did not match inclusion criteria.

Dolan J, Dolan Looby SE. Determinants of nurses’ use of physical restraints in surgical intensive care unit patients. Am J Crit Care 2017, 26: 373–379. Reason for exclusion: The context of this study did not match the inclusion criteria.

Karaka T, Aydin ozkan S, Derya ister E. Physical restraint use in elderly patients: perceptions of nurses in university hospitals. Turk Geriatr Derg. 2018;21(4):588–95. Reason for exclusion: Lack of principles of PR use for hospitalized elderly people in the context of this study.

Jiang H, Li C, Gu Y, He Y. Nurses’ perceptions and practice of physical
restraint in China. Nurs Ethics. 2015;22(6):652–60. Reason for exclusion: Lack of principles of PR use for hospitalized elderly people in the context of this study.

Kong EH, Choi H, Evans LK. Staff Perceptions of Barriers to Physical Restraint-Reduction in Long-Term Care: A meta-synthesis. J Clin Nurs 2017;26(1-2):49-60. Reason for exclusion: Lack of principles of PR use for hospitalized elderly people in the context of this study.

Krüger C, Mayer H, Haastert B and Meyer G. Use of physical restraints in acute hospitals in Germany: A multi-centre crosssectional study. Int J Nurs Stud 2013; 50: 1599–1606. Reason for exclusion: The context of this study did not match the inclusion criteria.

Kwok T, Bai X, Chui MY, Lai CK, Ho DW, Ho FK, Woo J. Effect of physical restraint reduction on older patients' hospital length of stay. J Am Med Dir Assoc. 2012;13(7):645-50. Reason for exclusion: Lack of principles of PR use for hospitalized elderly people in the context of this study.

Lam K, Kwan JSK, Wai Kwan C, Chong AML, Lai CKY, Lou VWQ, Leung AYM, Liu JYW, Bai X, Chi I. [Factors associated with the trend of physical and chemical restraint use among long-term care facility residents in Hong Kong: Data From an 11-Year Observational Study.](https://pubmed.ncbi.nlm.nih.gov/28843527/) J Am Med Dir Assoc. 2017;18(12):1043-1048. Reason for exclusion: The context of this study did not match the inclusion criteria.

Liverpool Hospital. Physical Restraints. (Intensive care unit: clinical guideline). Sydney (AU): NSW Government Health, South Western Sydney Local Health District; 2015: https://www.aci.health.nsw.gov.au/data/assets/pdf_file/0019/306451/ liverpoolPhysical. Reason for exclusion: Target group did not match inclusion criteria.

Johnson K, Curry V, Steubing A, Diana S, McCray A, McFarren A, Domb A. A non-pharmacologic approach to decrease restraint use. Intensive Crit Care Nurs. 2016;34:12-9. Reason for exclusion: The context of this document did not match the inclusion criteria.

Gu T, Wang X, Deng N, Weng W. [Investigating influencing factors of physical restraint use in China intensive care units: A prospective, cross-sectional, observational study.](https://pubmed.ncbi.nlm.nih.gov/30001953/) Aust Crit Care. 2019 May;32(3):193-198. Reason for exclusion: The objective of this study did not match review objective.

Hoeck S, François G, Geerts J, Van der Heyden J, Vandewoude M, Van Hal G. Health-care and home-care utilization among frail elderly persons in Belgium. Eur J Public Health. 2012;22(5):671-7. Reason for exclusion: The context of this document did not match the inclusion criteria.

Freeman S, Spirgiene L, Martin-Khan M, Hirdes JP. Relationship between restraint use, engagement in social activity, and decline in cognitive status among residents newly admitted to long-term care facilities. Geriatr Gerontol Int. 2017 Feb;17(2):246-255. Reason for exclusion: The context of this document did not match the inclusion criteria.

Lee H, Park M, Lee SH, Lee M, Go Y, Kim CG. Factors related to use of physical restraints for older adults with dementia in long-term care settings. J Korean Gerontol Nurs. 2019;21(2):125–34. Reason for exclusion: The language of the study did not match the inclusion criteria. Only the title and abstract were in English.

Freeman S, Hallett C, McHugh G. Physical restraint: experiences, attitudes and opinions of adult intensive care unit nurses. Nurs Crit Care 2016, 21:78–87. Reason for exclusion: The objective of this study did not match review objective.

[Maker](https://scholar.google.com/citations?user=Zfz6xrIAAAAJ&hl=en&oi=sra)Y, McSherry B. [Regulating restraint use in mental health and aged care settings: Lessons from the Oakden scandal](https://journals.sagepub.com/doi/abs/10.1177/1037969X18817592). Alternative Law Journal
2019, 44(1) 29–36. Reason for exclusion: Setting and target group did not match the inclusion criteria.

McCabe DE, Alvarez CD, McNulty SR, Fitzpatrick JJ. Perceptions of physical restraints use in the elderly among registered nurses and nurse assistants in a single acute care hospital. Geriatr Nurs. 2011;32(1):39–45. Reason for exclusion: Lack of principles of PR use for hospitalized elderly people in the context of this study.

Mohler R, Meyer G. Attitudes of nurses towards the use of physical restraints in geriatric care: a systematic review of qualitative and quantitative studies. Int J Nurs Stud. 2014;51(2):274–88. Reason for exclusion: lack of principles of PR use for hospitalized elderly people in the context of this study.

Möhler R, Richter T, Köpke S, Meyer G. Interventions for preventing and reducing the use of physical restraints in long-term geriatric care – a Cochrane review. J Clin Nurs. 2012;21:3070–81. Reason for exclusion: The setting of the study did not match the inclusion criteria.

Luk E, Burry L, Rezaie S, Mehta S, Rose L. [Critical care nurses' decisions regarding physical restraints in two Canadian ICUs: A prospective observational study.](https://pubmed.ncbi.nlm.nih.gov/26837121/) Can J Crit Care Nurs. 2015;26(4):16-22. Reason for exclusion: The context of this document did not match the inclusion criteria.

Saarnio R, Isola A. Nursing staff perceptions of the use of physical restraint
in institutional care of older people in Finland. J Clin Nurs. 2010;19(21–22):3197–207. Reason for exclusion: Lack of principles of PR use for hospitalized elderly people in the context of this study.

Leahy-warren P, Varghese V, Day MR, Curtin M. Physical restraint: perceptions of nurse managers, registered nurses and health care assistants. Int Nurs Rev. 2018;65(3):327–35. Reason for exclusion: The context of the document did not match the inclusion criteria.

Gheidari Z, Adib M, Mohamadii T, Kazemnejad E. Knowledge, attitude and performance of nurses in intensive care units in the field of patients’ physical restraint use and their related factors. J Adv Pharm Edu Res 2019;9(S2):109-114. Reason for exclusion: The objective of this study did not match review objective.

Heinze C, Dassen T, Grittner U. Use of physical restraints in nursing homes
and hospitals and related factors: a cross-sectional study. J Clin Nurs. 2012;
21:1033–40. Reason for exclusion: The context of the document did not match the inclusion criteria.

Lai CKY, Chow, SKY., Suen, LKP, Wong IYC. Reduction of physical restraints on patients during hospitalisation/rehabilitation: A clinical trial. Asian Journal of Gerontology and Geriatrics 2013, 8, 38-43. Reason for exclusion: Lack of principles of PR use for hospitalized elderly people in the context of this study.

Cosper P, Morelock V and Provine B. Please Release Me: restraint reduction initiative in a health care system. J Nurs Care Qual 2015; 30: 16–23. Reason for exclusion: Target group did not match inclusion criteria.

Möhler R, Nürnberger C, Abraham J, Köpke S, Meyer G. Interventions for
preventing and reducing the use of physical restraints of older people in
general hospital settings. Cochrane Database Syst Rev. 2016;2016(1212):
CD012476. Reason for exclusion: This was a protocol for a Cochrane Review and lack of principles of PR use for hospitalized elderly people.

Moradimajd P, Noghabi AA, Zolfaghari M, Mehran A. Physical restraint use
in intensive care units. Iran J Crit Care Nurs. 2015;8(3):173–8. Reason for exclusion: the context of the study did not match the inclusion criteria.

Pellfolk T, Sandman PO, Gustafson Y, Karlsson S, Lovheim H. [Physical restraint use in institutional care of old people in Sweden in 2000 and 2007](https://www.cambridge.org/core/journals/international-psychogeriatrics/article/physical-restraint-use-in-institutional-care-of-old-people-in-sweden-in-2000-and-2007/43F75575A7E652564C7367480283F2C3). International Psychogeriatrics 2012, 24:7, 1144–1152. Reason for exclusion: Lack of principles of PR use for hospitalized elderly people in the context of this study.

Raveesh BN, Gowda GS, Gowda M. Alternatives to use of restraint: A path toward humanistic care. Indian J Psychiatry. 2019;61(l 4):S693-S697. Reason for exclusion: Setting and target group of patients did not match inclusion criteria.

Saeidi S, Khatiban M, Khazaei A, Soltanian A, Rahimi-Bashar F. Assessment of intensive care unit nurses’ knowledge, attitude, and practice of physical restraint use. Sci J Hamadan Nurs Midwifery Fac. 2015;23(3):40–9. (Persian) Reason for exclusion: Target group did not match inclusion criteria.

Elhameed SHA, Elemam NI. Nurses knowledge, intention and attitude towards the use of physical restraintin geriatric care. IOSR Journal of Nursing and Health Science (IOSR-JNHS), 9(01), 2020, 30-38. Reason for exclusion: Lack of principles of PR use for hospitalized elderly people in the context of this study.

Muir-Cochrane E, Baird J, McCann T. Nurses’ experiences of restraint and
seclusion use in short-stay acute old age psychiatry inpatient units: a
qualitative study. J Psychiatr Ment Health Nurs. 2015;22(2):109–15. Reason for exclusion: the context of the study did not match the inclusion criteria.

Wang J, Liu W, Peng D, Xiao M, Zhao Q. The use of physical restraints in Chinese long-term care facilities and its risk factors: An observational and cross sectional study. J Adv Nurs. 2020;76:2597–2609. Reason for exclusion: the context of the study did not match the inclusion criteria.

NICE Guidelines on Violence and Aggression May 2015:
<https://www.nice.org.uk/guidance/ng10>. Reason for exclusion: The context of the document did not match the inclusion criteria.

Luk E, Sneyers B, Rose L, Perreault MM, Williamson DR, Mehta S, Burry L. Predictors of physical restraint use in Canadian intensive care units. Crit Care. 2014;18(2):R46. Reason for exclusion: The context of this study did not match the inclusion criteria.

Perkins E, Prosser E, Riley D, et al. Physical restraint in a therapeutic setting: a necessary evil. Int J Law Psychiatry 2012; 35: 43–49. Reason for exclusion: The context of this study did not match the inclusion criteria.

Stevens JC. The use of physical restraints in neurologic patients in the inpatient setting. Continuum 2012; 18(6): 1422–1426. Reason for exclusion: The context of this study did not match the inclusion criteria.

Rose L, Dale C, Smith OM, Burry L, Enright G, Fergusson D, et al. A mixedmethods systematic review protocol to examine the use of physical
restraint with critically ill adults and strategies for minimizing their use. Syst
Rev. 2016;5(1):194. Reason for exclusion: This was a systematic review protocol and did not match the inclusion criteria.

Strategies to end the use of seclusion, restraint and other coercive practices - WHO
QualityRights training to act, unite and empower for mental health (pilot version). Geneva: World Health Organization; 2017 (WHO/MSD/MHP/17.9). Licence: CC BY-NC-SA 3.0 IGO. Reason for exclusion: The context of this document did not match the inclusion criteria.

Yevchak A, Fick DM, Kolanowski AM, McDowell J, Monroe T, LeViere A, Mion L. Implementing nurse-facilitated person-centered care approaches for patients with delirium superimposed on dementia in the acute care setting. J Gerontol Nurs. 2017 Dec 1;43(12):21-28. Reason for exclusion: The context of this document did not match the inclusion criteria.

Van der Kooi AW, Peelen LM, Raijmakers RJ, Vroegop RL, Bakker DF, Tekatli H, van den Boogaard M, Slooter AJ. Use of physical restraints in Dutch intensive care units: a prospective multicenter study. Am J Crit Care. 2015 Nov;24(6):488-95. Reason for exclusion:The objective of this study did not match review objective.

Comprehensive guide to national accreditation of Iranian hospitals; Edited in 2016**.** <http://ghhospital.mui.ac.ir/sites/ghhospital.mui.ac.ir/files/etb1pdf>. (Persian) Reason for exclusion: Lack of principles of PR use for hospitalized elderly people in the context of this study.

Andrade MRS, Couto MB, Carvalho ACS, Barros PFA, Delvalle R, Santana RF. Perception of a multidisciplinary team on physical restraint use on older adults: care paradoxes. Geriatr Gerontol Aging. 2020;14(3):181-8. Reason for exclusion: Setting did not match the inclusion criteria. Lack of principles of PR use for hospitalized elderly people in the context of this study.
