# Supplementary material for: The principles of physical restraint use for hospitalized elderly people: an integrated literature review
Source: Syst Rev. 2021 May 1;10:129. doi: 10.1186/s13643-021-01676-8 (PMC8088072; doi:10.1186/s13643-021-01676-8)
Supplement: Supplementary file 4 — Additional file 4. Characteristics of the included documents. The characteristics of documents included in the present study. [file 13643_2021_1676_MOESM4_ESM.docx]

**Additional file 4.** Characteristics of the included documents. The characteristics of documents included in the present study.

| **No.** | **First author/Organization** | **Year** | **Country** | **Document type/Purpose** | **Quality appraisal** | |
| --- | --- | --- | --- | --- | --- | --- |
|  |  |  |  |  | **JBI^a^** | **AGREE II^b^** |
| 1 | Agens/- [[42](#_ENREF_40)] | 2010 | United States | Theoretical (brief review)/Assessing chemical and physical restraint use for elderly people | 5/6 |  |
| 2 | Dikiciyan/Federal Council of the Australian and New Zealand Society for Geriatric Medicine [[10](#_ENREF_7)] | 2016 | Australia and New Zealand | Theoretical (statement)/Describing the use of PR for elderly people | 4/6 |  |
| 3 | Mcneill et al. /Registered Nurses’ Association of Ontario [[35](#_ENREF_38)] | 2016 | Canada | Guideline/Delirium, dementia, and depression assessment and care among elderly people |  | 7/7 |
| 4 | -/Health Service Executive [[37](#_ENREF_38)] | 2011 | Ireland | Theoretical (policy)/Minimizing the complications of PR use for elderly people | 5/6 |  |
| 5 | Said et al./- [[9](#_ENREF_6)] | 2013 | United States | Theoretical (brief review)/Reviewing restraint-related consequences and providing strategies to reduce PR use for elderly people | 5/6 |  |
| 6 | Lachance et al./Canadian Agency for Drugs and Technologies in Health [[43](#_ENREF_35)] | 2019 | Canada | Theoretical (report)/Reviewing clinical effectiveness and evidence-based guidelines related to PR use for hospitalized elderly people | 5/6 |  |
| 7 | Cotter et al./Hartford Institute for Geriatric Nursing [[8](#_ENREF_5)] | 2018 | United States | Theoretical (statement)/Avoiding restraint in hospitalized elderly people | 6/6 |  |
| 8 | -/American Academy of Nursing [[30](#_ENREF_36)] | 2014 | United States | Theoretical (statement)/Choosing wisely PR in hospitalized elderly people | 5/6 |  |
| 9 | Peisah et al./- [[52](#_ENREF_41)] | 2011 | Australia | Guideline/Practical guidelines for the management of agitated older patient |  | 4/7 |
| 10 | -/American Geriatrics Society [[53](#_ENREF_41)] | 2015 | United States | Theoretical (expert opinion) /Recommendations for PR use for hospitalized elderly people | 4/6 |  |
| 11 | Snook et al./Cinahl Information Systems [[38](#_ENREF_43)] | 2017 | United States | Theoretical (clinical review) /Reviewing the interventions to reduce the use of PR | 5/6 |  |
| 12 | Cleary et al./- [[32](#_ENREF_25)] | 2015 | United States | Theoretical (brief review)/Reviewing the regulations, ethical implications, and legal considerations related to PR use | 5/6 |  |
| 13 | -/American Nurses Association [[11](#_ENREF_8)] | 2012 | United States | Theoretical (statement)/Addressing the role of registered nurses in reducing restraint use | 5/6 |  |
| 14 | Wagner et al./Registered Nurses’ Association of Ontario [[36](#_ENREF_43)] | 2012 | Canada | Guideline/Evidence-based best practices and strategies for assessment, prevention, and use of restraint |  | 7/7 |
| 15 | Zencirci/- [[12](#_ENREF_9)] | 2012 | Turkey | Theoretical (discussion paper) /Providing a guide for good practice in PR use | 5/6 |  |
| 16 | Lach et al./Center of Gerontological Nursing Excellence [[18](#_ENREF_26)] | 2016 | United States | Guideline/Guideline on how to reduce PR use |  | 4/7 |
| 17 | Lim et al./- [[7](#_ENREF_4)] | 2016 | Singapore | Theoretical (discussion paper) /Restraint use in the management of hospitalized elderly people | 5/6 |  |
| 18 | Hzzard et al./- [[13](#_ENREF_10)] | 2017 | United States | Theoretical (E-book)/ Managing delirium risk among hospitalized elderly people | 6/6 |  |
| 19 | Flaherty et al./- [[54](#_ENREF_10)] | 2011 | United States | Theoretical (discussion paper) /Matching the Restraint-Free Environment for Older Hospitalized Adults with Delirium. | 5/6 |  |
| 20 | -/American Family Physician [[55](#_ENREF_10)] | 2020 | United States | Theoretical (expert consensus) /Choosing Wisely managing behavioral symptoms of hospitalized older adults with delirium | 5/6 |  |

**a:** JBI Critical Appraisal Checklist for Text And Opinion Papers consists of 6 items with 4 answer possibilities [48].

**b:** AGREE II consists of 23 items divided over 6 domains. Each item is rated on a 7-point scale ranging from strongly disagree (1) to strongly agree (7). The assessment is based on the total score of the 23 items and whether the user wants to recommend the guideline for use. Rate the overall quality of this guideline: 1 (lowest possible quality) – 7 (highest possible quality) [49].
